# A Denoising Preprocessing Framework via Orthogonal Multi-Tap Null-Steering Beamformer Bank: Facilitating Target Signal Preservation Under Low SINR Conditions and Complex Soundscapes

**DOI:** 10.3390/s26103186

**Published:** 2026-05-18

**Authors:** Lei Chen, Zhiyong Xu, Pukun Su, Zhao Zhao

**Affiliations:** School of Electronic and Optical Engineering, Nanjing University of Science and Technology, Nanjing 210094, China; clei@njust.edu.cn (L.C.); 123104222567@njust.edu.cn (P.S.); zhaozhao@njust.edu.cn (Z.Z.)

**Keywords:** differential microphone array, null-steering beamformer bank, acoustic indices, passive acoustic monitoring, interference suppression, target signal self-cancellation

## Abstract

Acoustic indices are popular tools for rapid biodiversity assessment using passive acoustic monitoring recordings, yet anthropogenic sounds in human activity areas compromise their robustness. In this paper, we focus on the typical urban–rural soundscape, where anthropogenic noise mainly originates from a narrow angular sector far from the monitoring device. We propose a denoising preprocessing algorithm with two microphone sensors for the robust application of existing acoustic indices. Our algorithm first develops an adaptive multi-tap null-steering beamformer based on a back-to-back first-order differential microphone array, which increases the system degrees of freedom to enhance the broadband interference cancellation capability. Building on this, a parallel bank of mutually orthogonal null-steering beamformers is proposed, each forming deep nulls toward directional interference-concentrated bands and generating diverse responses to the target signal. Finally, a signal compensation mechanism is applied to the beamformers’ outputs, mitigating the signal self-cancellation effects from these unconstrained adaptive beamformers prior to index calculation. The proposed preprocessing method is evaluated using the frequency-dependent acoustic diversity index as a representative of acoustic indices. Experiment results on both simulation and real-world recordings show that the proposed method generates high-fidelity acoustic information for subsequent acoustic index calculation over a much wider signal-to-interference-plus-noise ratio (SINR) range in urban–rural soundscapes characterized by directional anthropogenic interference.

## 1. Introduction

Rapid and reliable biodiversity assessment has become an increasingly urgent mission in the context of global environmental change [1,2]. Despite their accuracy, traditional large-scale field surveys incur substantial temporal and financial expenditures as well as have inherent observer bias [1,2,3,4]. In recent years, passive acoustic monitoring (PAM) offers a promising alternative for long-term and large-scale biodiversity assessment [5,6]. By continuously recording soundscapes, PAM enables automated and non-invasive observation of wildlife activities across large spatial and temporal scales [2,3,4], facilitating comprehensive ecological monitoring.

With the PAM framework, acoustic indices have attracted increasing attention due to their potential for rapid biodiversity assessment (RBA) [1,3,4]. These indices summarize recordings and characterize ecosystems efficiently. It is worth noting that this approach shifts the focus from species-level identification to holistic rapid assessments of acoustic communities, focusing on scenarios involving multiple bird species. However, their practical application faces significant challenges, as existing acoustic indices are significantly affected by noise [3,4,7,8]. This limitation is particularly critical in human-dominated soundscapes, such as urban–rural areas. With the growing demand for comprehensive environmental monitoring [9], RBA is gradually expanding to complex soundscapes dominated by anthropogenic and geophonic sounds. These non-biotic signals overlap with biotic sounds in both time and frequency, leading to biased or even controversial ecological interpretations among different studies [8]. For clarity and ease of preprocessing, we terminologically classify anthropogenic sounds as interference due to their deterministic nature and spatio-temporal correlation. In contrast, geophonic sounds from wind and rain, along with other spatial uncorrelated additive noise in recordings, are still termed noise.

Conventional preprocessing for acoustic indices relies on high-pass filtering to remove low-frequency components [1,7,10,11]. This strategy inherently assumes spectral separability between non-biotic and biotic sounds, an assumption that fails in urban–rural soundscapes where the broadband interference overlaps with biotic signals in frequency. For example, the conventional acoustic diversity index (ADI [12]) uses a single dBFS threshold across the entire time–frequency (TF) space in analysis. Previous studies have shown that the high-pass filter alone cannot suppress the adverse impact of non-biotic sound on ADI [4,10,11]. Recently, the frequency-dependent ADI (FADI) variant [4] was proposed, in which floating thresholds adapted to the noise level at each frequency bin were employed, with the single dBFS threshold of ADI serving as the lower limit. This design introduced an individualized detection threshold for each frequency bin based on its narrowband noise level, which can effectively filter out much of the temporally steady noise and interference. However, the local signal-to-interference-plus-noise ratio (SINR) is often quite low in interference-concentrated frequency bands, where the biotic sound TF points masked by interference cannot pass the threshold detection, resulting in distorted FADI results [13]. Clearly, existing single-channel noise suppression methods, including both high-pass filter preprocessing or frequency-dependent threshold detection, cannot guarantee reliable applications of ADI and its variants in the presence of strong interferences. The adoption of microphone array processing technology with multi-channel spatial filtering capabilities [14,15,16,17,18,19] represents a necessary evolution.

**Figure 1:**
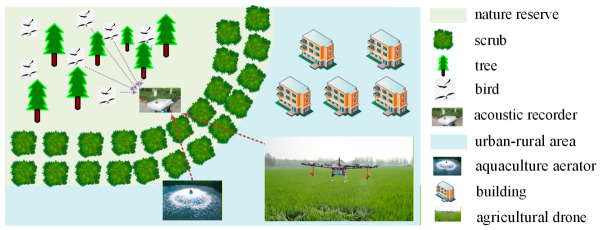
Typical geometric relationship of biodiversity monitoring in urban–rural areas.

In RBA applications, the deployment of acoustic recording devices is carefully selected to maximize the bird sounds and minimize the impact of anthropogenic interference on index calculations. Even in urban–rural areas where anthropogenic noise is unavoidable, devices are deployed at locations away from human activity zones that are characterized by relatively single-source interference direction. In such cases, although the field audio recordings include both avian vocalizations and anthropogenic interferences, the recordings contain at most a single dominant interference. Therefore, this paper does not consider complex scenarios with multiple dispersed interferences. For moving interferences in the area, their angular variation range is typically limited to a relatively narrow sector due to their remote distance from the recording device. Figure 1 illustrates a typical geometric relationship of biodiversity monitoring in urban–rural areas, where sounds from agricultural machinery are the typical interferences. These interferers are not continuously active throughout the year; instead, they may operate persistently for several consecutive days, particularly during farming or harvest seasons. During such active periods, the soundscape typically comprises vocalizations from multiple bird species alongside a single directional interference source. Inevitably, acoustic indices will be inaccurate since biotic sounds are masked by the interference. Notably, these interferences exhibit significant spatio-temporal correlation, referred to in this paper as directional interference signals. In this context, the approximate directions of the interferences are either a priori known or readily measurable. Therefore, to achieve continuous monitoring, a microphone array processing strategy with deep interference suppression is required before the index calculation.

However, the calculation of acoustic indices requires capturing avian vocalizations across the entire spatial domain as completely as possible while suppressing interference. This implies that the mainstream microphone array beamforming techniques characterized by directional enhancement of a narrow beam are no longer suitable. Instead, a first-order differential microphone array (FO-DMA) [20,21,22] using two close-spaced microphone sensors is more appropriate for the targeted urban–rural soundscapes characterized by strong but directional interference, because of its unique spatial null filtering response and low-cost, simple implementation. Specifically, its cardioid pattern provides a broad coverage beam with a sharp on-axis null for interference cancellation. In addition, FO-DMA inherently possesses a first-order differentiator frequency response that can suppress low-frequency noise, particularly the impact of equipment self-noise on subsequent exponential calculations.

Since interference directions are either known a priori or tracked in real time via established direction of arrival (DOA) estimation methods [23,24,25,26,27], an FO-DMA can theoretically steer its cardioid pattern’s null precisely toward the interference direction by mounting on a turntable setup. Nevertheless, various non-ideal factors (such as DOA estimation errors and sound speed variations) may cause misalignment between the null center and the actual interference direction in practice. Obviously, a fixed cardioid beam cannot ensure the required interference suppression performance for acoustic index applications, necessitating an adaptive FO-DMA algorithm to dynamically adjust its null position to achieve consistent cancellation of directional interference within the rear half-space. Note that combining the two FO-DMA elements can simultaneously form two back-to-back cardioid beams with identical patterns but opposite look directions. If a directional interference has a small offset angle from the null direction of the forward-beam, its residual component in the forward-beam output can be estimated by applying optimal weighting to the newly added backward-beam signal. This estimate is then subtracted from the forward-beam output to make the rear null of the array pattern align with the true interference direction [28]. However, traditional adaptive null-steering based on FO-DMA, which uses a Wiener filter of length one [28], can only form frequency-dependent nulls due to the limited system degrees of freedom. As a result, the spatial null aligns with the interference direction solely within the band of peak interference energy, deviating at other frequencies. This lack of the capability for broadband interference cancellation will bias the subsequent index calculation.

Building upon the previous discussion, this paper proposes a novel transferable denoising preprocessing framework to facilitate broader adoption of acoustic indices for biodiversity monitoring in complex urban–rural soundscapes. Leveraging the orthogonality between the noise subspace and the interference subspace, as well as the geometric uncertainty between the noise and signal subspaces, this framework extracts multiple mutually orthogonal eigenvectors from the noise subspace to formulate the multi-tap null-steering beamformer weights for each parallel channel. Each channel can form deep narrow groove along the interference’s spatio-temporal support while exhibiting diverse responses to the target signals. As a result, the framework effectively suppresses interference while preserving target acoustic information to the greatest extent possible through the fusion of multi-channel outputs. Experimental results demonstrate that our proposed method leverages elaborate signal processing stages before index calculation, ensuring numerical robustness of index values under low SINR conditions and complex soundscapes. The primary contributions of this work are as follows:

(1) A multi-tap spatio-temporal null-steering beamformer based on the back-to-back FO-DMA structure is proposed, which provides more system degrees of freedom for broadband interference suppression. In contrast to the conventional null-steering beamformer, this multi-tap approach forms the narrow groove in the interference’s spatio-temporal support, significantly enhancing the broadband interference cancellation capability.
(2) A bank of mutually orthogonal null-steering beamformers combined with a signal compensation algorithm is proposed to mitigate the self-cancellation of the target signal from the unconstrained filtering process. Compared with the single beamformer scheme, the multi-branch design effectively preserves the desired signal while suppressing interference within a wider SINR range.
(3) The two-element FO-DMA employed in our method features a simple structure, compact size, and low complexity. This enables low power, low-cost implementation, making it ideal for large-scale deployment and long-term biodiversity monitoring. Consequently, it can substantially expand the spatio-temporal coverage of acoustic sensing.

The rest of this paper is organized as follows. Section 2 introduces the proposed denoising preprocessing algorithm. Experimental results using both simulation and real-world recordings are reported in Section 3. Section 4 presents further discussions and Section 5 draws the conclusions.

## 2. Materials and Methods

**Figure 2:**
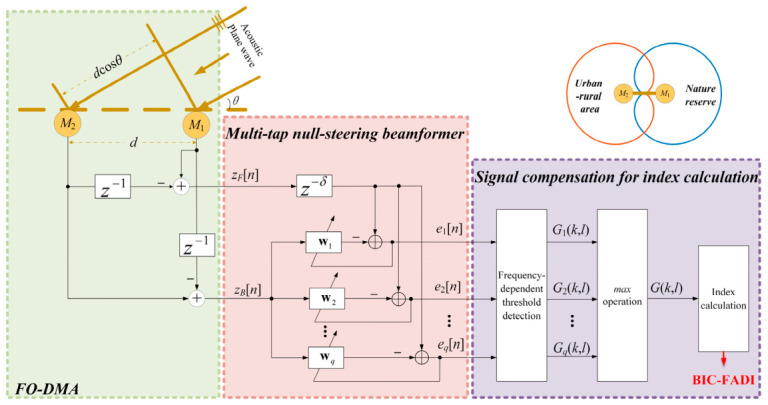
Graphical illustration of the proposed system. The green FO-DMA part collects the noisy signal. The pink parallel beamformer bank part performs denoising preprocessing to suppress anthropogenic noise. The purple signal compensation for index calculation part is given using FADI as an example.

The framework of the proposed denoising preprocessing algorithm for robust application of acoustic indices is depicted in Figure 2, which consists of three key parts: (1) the microphone array configuration; (2) the denoising preprocessing method; (3) the signal compensation for index calculation. Initially, the noisy signal collected by an acoustic recording device with two microphone sensors is mathematically modeled and analyzed. The noisy signal is then preprocessed using a bank of mutually orthogonal null-steering beamformers, each forming deep nulls toward directional interference-concentrated bands. Finally, a compensation algorithm applying point-wise logical OR fusion on the output binary spectrograms is employed to mitigate signal self-cancellation effects from these unconstrained adaptive beamformers prior to index calculation. In this paper, we take FADI to serve as a representative of acoustic indices to validate the proposed preprocessing algorithm. For clarity, the index calculated after applying the proposed denoising preprocessing method is denoted as backward interference cancellation—FADI (BIC-FADI).

### 2.1. Structure of the FO-DMA

The discrete noisy signal collected by an acoustic recording device is

(1) $$x\left[n\right]=s\left[n\right]+v\left[n\right]=s\left[n\right]+\phi \left[n\right]+\varphi \left[n\right],$$

where n is the discrete-time index, $s\left[n\right]$ represents the biotic sound signal, and $v\left[n\right]=\phi \left[n\right]+\varphi \left[n\right]$ is the interference-plus-noise component, which is collectively referred to as noise in existing literature on acoustic indices. For an urban–rural area, strong interference sources typically exist in a small and known angular range. Therefore, the interference signal $\phi \left[n\right]$ has strong spatial correlation, whereas the noise $\varphi \left[n\right]$ is spatially uncorrelated.

**Figure 3:**
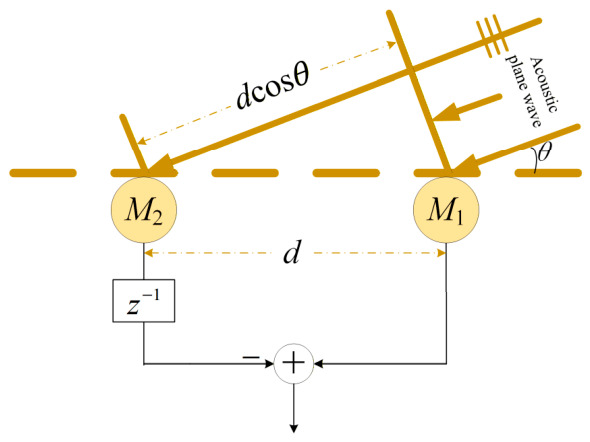
Diagram of the first-order differential microphone array.

A nearly omnidirectional null-steering beamformer with one narrow spatial null is suitable to suppress the directional interference while preserving as much avian vocalization information as possible from all non-interfering directions. Such a beamformer can be realized with an FO-DMA, which is composed of two close-spaced omnidirectional microphone sensors and a delay unit after one of them. As is illustrated in Figure 3, the output of the FO-DMA is obtained by subtracting the previous sample of $M_{2}$ channel from the current sample of $M_{1}$ channel.

Given an acoustic far-field plane wave incident on the array at an arbitrary angle of θ (counter-clockwise is positive), the array endfire direction ($M_{2}\to M_{1}$) is defined as 0°. When the two received signals have a negligible amplitude difference and a time difference of τ=−dcosθ/c between them (with $M_{1}$ as a reference), we have $x_{1}\left[n\right]=x_{2}\left[n-\tau /T_{s}\right]$, where d, c, and $T_{s}$ are the array spacing, the speed of sound in air, and the sampling period, respectively. Let $\rho =(d/c)/T_{s}$ be the actual ratio of the external to internal delays, which is equal to 1 in an ideal case. Then the array output can be expressed as

(2) $$z_{F}\left[n\right]=x_{1}\left[n\right]-x_{2}\left[n-1\right]=x_{2}\left[n+\rho \cos\theta \right]-x_{2}\left[n-1\right],$$

whose spatio-temporal amplitude response is

(3) $$H_{F}(f,\theta )=\left|\frac{Z_{F}(f,\theta )}{X_{2}(f)}\right|=\left|2\sin\frac{\pi f\left(1+\rho \cos\theta \right)}{f_{s}}\right|,$$

where $Z_{F}(f,\theta )$, $X_{2}(f)$ are the spectra of array output $z_{F}\left[n\right]$ and signal $x_{2}\left[n\right]$, respectively. $f_{s}=1/T_{s}$ is the sampling rate.

**Figure 4:**
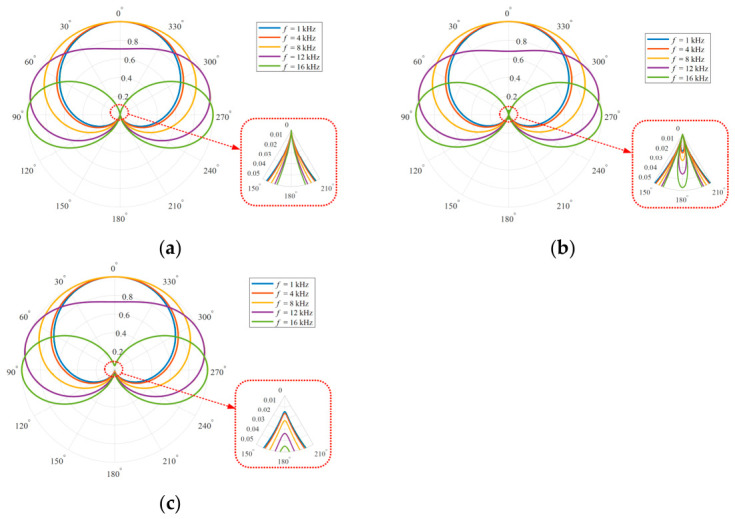
The array directivity patterns at different frequencies when $f_{s}=32\;\mathrm{kHz}$. The red box highlights a detailed view of the frequency-independent null. (**a**) ρ=1; (**b**) ρ>1; (**c**) ρ<1.

In practical applications, the sampling period (i.e., the internal delay) $T_{s}$ is selected according to the spectral distribution of the signal-of-interest, and the element spacing d is a fixed system parameter that is configured under the ideal case of ρ=1 with a typical value of sound speed c (e.g., c=340 m/s in this paper). However, as c is a complicated function of multiple environmental factors including temperature, humidity, and pressure, ρ definitely deviates from 1 most of the time due to the time-varying nature of c. Figure 4 presents the array directivity patterns at different frequencies (when $f_{s}=32\;\mathrm{kHz}$) for the case of ρ=1, ρ>1, and ρ<1 according to Equation (3).

Figure 4 illustrates the array directivity patterns with different ρ values at frequencies of 1 kHz, 4 kHz, 8 kHz, 12 kHz, and 16 kHz when $f_{s}=32\;\mathrm{kHz}$. It shows that the FO-DMA forms one frequency-independent null at every frequency. Nonetheless, both the position and depth of this null depend on ρ. The null is located at 180° only in the ideal case (i.e., ρ=1). It splits when ρ>1 or becomes shallower when ρ<1, impairing the nulling capability to different extents at different frequencies. This implies that even if the design null of the ideal cardioid pattern could exactly align with the interference direction, there will still be a considerable interference residual in the array output. In addition, other impairment factors such as biased null steering due to incorrect direction estimation or sound source motion will further degrade interference suppression performance.

In order to achieve robust interference suppression, an adaptive null-steering beamformer based on back-to-back FO-DMA was proposed in [28]. This method utilizes the symmetry of a two-element microphone array to simultaneously generate a pair of cardioid patterns with diametrically opposed directivities. For a far-field signal arriving from a direction around 180°, which is in the rear half-plane of the forward-beam defined in Equation (2) (denoted with subscript *F*), the output of the backward-beam is

(4) $$z_{B}\left[n\right]=x_{2}\left[n\right]-x_{1}\left[n-1\right]=x_{2}\left[n\right]-x_{2}\left[n+\rho \cos\theta -1\right],$$

and the corresponding spatio-temporal amplitude response is

(5) $$H_{B}(f,\theta )=\left|\frac{Z_{B}(f,\theta )}{X_{2}(f)}\right|=\left|2\sin\frac{\pi f\left(\rho \cos\theta -1\right)}{f_{s}}\right|,$$

where $Z_{B}(f,\theta )$ represents the spectrum of the output of the backward-beam output $z_{B}\left[n\right]$.

**Figure 5:**
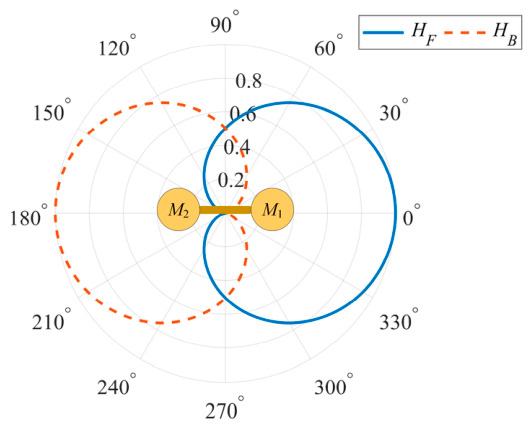
Ideal beam patterns of the back-to-back FO-DMA under the ideal condition. The blue solid line and the red dashed line represent the forward and backward beam patterns, respectively.

Figure 5 presents an example of the dual back-to-back cardioid beams based on Equations (3) and (5) under the ideal condition of ρ=1. The directivity of both cardioids is mirrored with respect to the array’s normal direction. In the application of acoustic indices for urban–rural soundscapes, the array orientation can be mechanically steerable to make the interference direction lie within the forward-beam’s null-width. In such a case, the backward-beam captures the enhanced interference signal, and then the interference residual in the forward-beam can be removed by an adaptive filtering process that is introduced in Section 2.2.

### 2.2. Realization of Space-Time Null-Steering Beamforming

#### 2.2.1. Back-to-Back FO-DMA-Based Null-Steering Beamformer

**Figure 6:**
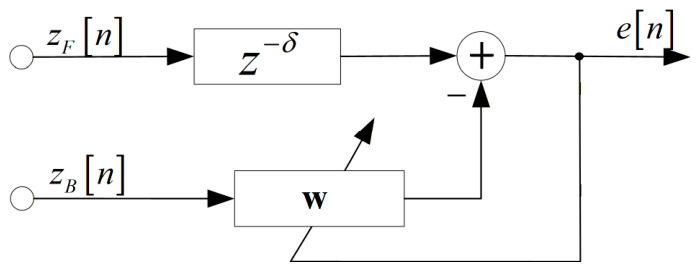
Diagram of a multi-tap null-steering beamformer.

To achieve deep suppression of directional interference while maximally preserving the TF distribution information of biotic sound signals, a parallel bank of multi-tap null-steering beamformers based on the back-to-back FO-DMA is proposed in this study. The structure of a multi-tap null-steering beamformer is shown in Figure 6. This beamformer employs the forward-beam as the main channel (the upper branch) and the backward-beam the auxiliary channel (the lower branch). It utilizes the weighted summation of the latest h samples in the backward-beam to estimate the δ-delayed sample value of the forward-beam and then outputs the subtraction result of both. Note that the filter length h and the delay δ satisfy h=2δ+1. The output of the multi-tap null-steering beamformer is

(6) $$e\left[n\right]=z_{F}\left[n-\delta \right]-w^{T}z_{B}\left[n\right],$$

where δ is an integer delay item, and $z_{B}\left[n\right]={\left[z_{B}\left[n\right],\;z_{B}\left[n-1\right],\;\ldots ,\;z_{B}\left[n-h+1\right]\right]}^{T}$ is a sample of input vector at time n, w denotes weighting vector used to calculate the estimate of the interference component in the forward-beam. The optimal weighting vector for minimizing the output power of interference is given by the Wiener–Hopf equation [29]

(7) $$w_{opt}=R_{v}^{-1}r_{v},$$

where $R_{v}=E\{z_{B}\left[n\right]z_{B}^{T}\left[n\right]\}$ is the lower branch correlation matrix, and $r_{v}=E\{z_{F}\left[n-\delta \right]z_{B}\left[n\right]\}$ is the cross-correlation between the upper and lower branches $z_{F}\left[n-\delta \right]$ and $z_{B}\left[n\right]$. E{⋅} represents the expectation operator.

Although this adaptive null-steering beamformer appears to follow a generalized sidelobe canceller (GSC) [30] architecture, it fundamentally differs from GSC due to significant main-beam overlap between the backward-beam and the forward-beam. This overlap inevitably introduces the self-cancellation of the target signal. To mitigate this, the adaptive weight for interference cancellation is estimated during the interference-dominated periods devoid of avian vocalizations, based on the prior assumption that the target signal and interference are mutually uncorrelated. Specifically, this paper adopts an offline processing framework. We conduct auditory checks to select interference-plus-noise segments devoid of any bird sounds, after which the samples within these segments are utilized to estimate the correlation matrix $R_{v}$ and $r_{v}$ [31,32]. Specifically, $R_{v}$ and $r_{v}$ are usually replaced by the sample covariance matrix ${\hat{R}}_{v}$ and ${\hat{r}}_{v}$,

(8) $${\hat{R}}_{v}=\frac{1}{Y}\sum_{n_{y}\in ɤ}z_{B}\left[n_{y}\right]z_{B}^{T}\left[n_{y}\right],$$

(9) $${\hat{r}}_{v}=\frac{1}{Y}\sum_{n_{y}\in ɤ}z_{F}\left[n_{y}-\delta \right]z_{B}\left[n_{y}\right],$$

where ɤ is the index set of interference-plus-noise samples, and Y denotes the total number of such samples (i.e., the sample support). A larger sample support generally improves the estimation accuracy of the correlation matrix for stationary data [32,33,34].

In a typical acoustic scene, the types of interference sources tend to remain stable. Based on this prior knowledge, the subspace dimension of interference can be estimated using principal component analysis (PCA) [35,36] before system deployment. This allows for setting an appropriate filter length for the aforementioned adaptive filter since the optimal filter length should slightly exceed the subspace dimension of interference. Such an approach prevents ill-conditioning from an excessively large filter length and avoids incomplete cancellation from a small filter length [37].

**Figure 7:**
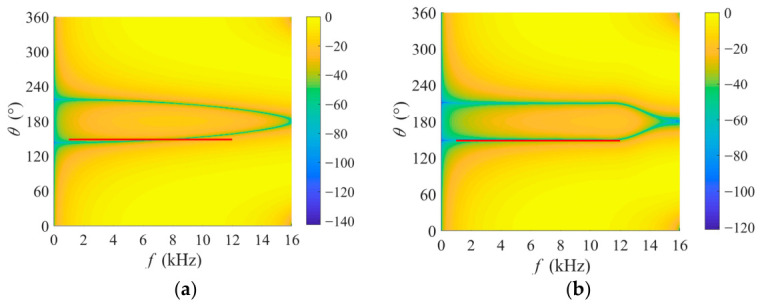
Normalized spatio-temporal amplitude responses of different filter lengths under an interference direction of 150°. The red solid line indicates the interference’s spatio-temporal support. (**a**) h = 1; (**b**) h = 21.

To further analyze the impact of filter length (i.e., system degree of freedom) on interference suppression performance, this paper presents the normalized spatio-temporal amplitude responses corresponding to various filter lengths. The broadband interference signal, spanning 1–12 kHz, is located at 150°. As shown in Figure 7, its spatio-temporal support is indicated by the red solid line. Figure 7a demonstrates that a single system degree of freedom is inadequate against the wideband interference, resulting in a frequency-dependent null positions rather than alignment with the interference direction across the entire interference band. Note that when the filter length is 1, the null-steering beamformer is the adaptive filtering process in [28]. For the broadband interference used in this experiment, the appropriate filter length is 21 according to the subspace dimension of interference. A comparison across subfigures reveals that as the filter length increases, the multi-tap nulling-steering beamformer forms a more continuous groove within the interference’s spatio-temporal support. This broadband interference cancellation capability is highly beneficial for subsequent acoustic index calculation. Moreover, Figure 7 also shows that the FO-DMA naturally exhibits a high-pass effect, which can effectively filter out low-frequency noise such as the equipment self-noise, further reducing its impact on the index calculations.

#### 2.2.2. The Parallel Beamformer Weights

The single weighting solution obtained by Equation (7) follows the principle of minimizing the output power, which lacks any explicit constraint to preserve the target signals. In fact, it is difficult to impose any effective constraints since bird vocalizations exhibit highly variable spectra and random DOAs. As a result, this method inevitably leads to severe self-cancellation of the target signal during the denoising process.

To address this issue, this paper proposes a parallel beamformer structure whose weights are mutually orthogonal. Leveraging the fundamental subspace property that the noise subspace is orthogonal to the interference subspace, we convert multiple, mutually orthogonal eigenvectors from the noise subspace into practical filter weights for each branch. Each eigenvector forms a separate filtering branch, forming deep nulls along the interference’s spatio-temporal support and generating diverse suppression patterns with the target signal subspace. The difference in self-cancellation across the branches forms the cornerstone for subsequent target signal compensation through multi-channel information fusion.

Specifically, Equation (6) can be reformulated as

(10) $$e\left[n\right]=\left[1-w^{T}\right]\left[\begin{array}{c} z_{F}\left[n-\delta \right]\\ z_{B}\left[n\right] \end{array}\right]=w_{FB}^{T}z_{FB},$$

and C is the $\left(h+1\right)\times \left(h+1\right)$ covariance matrix of $z_{FB}$. According to the Hermitian symmetry of the covariance matrix, its eigenvalue decomposition is

(11) $$C=U\Lambda U^{H}=\sum_{j=1}^{h+1}\lambda_{j}u_{j}u_{j}^{H},$$

where $U=\left[u_{1},u_{2},\cdots ,u_{h+1}\right]$ is a unitary matrix composed of orthonormal eigenvectors, and $\Lambda =\mathrm{diag}\left(\lambda_{1},\lambda_{2},\cdots ,\lambda_{r},\lambda_{r+1},\cdots ,\lambda_{h+1}\right)$ contains the corresponding eigenvalues in descending order. Therefore, the unitary matrix can be decomposed into $U=\left[U_{\phi }\;U_{\varphi }\right]$, where $U_{\phi }=\left[u_{1},u_{2},\ldots ,u_{r}\right]$ and $U_{\varphi }=\left[u_{r+1},u_{r+2},\ldots ,u_{h+1}\right]$ span the interference and noise subspaces, respectively. The parameter r denotes the dimension of the interference subspace, satisfying

(12) $$\sum_{j=1}^{r}\lambda_{j}\geq \zeta \sum_{i=1}^{h+1}\lambda_{i},$$

where ζ is usually set to 0.95 [35], i.e., 95% of the energy is retained.

Correspondingly, the dimension of the noise subspace is h+1−r. This paper uses the eigenvector corresponding to the smallest eigenvalue to calculate the weight for the first branch, i.e., the h+1-th eigenvector $u_{h+1}$. In general, the weight for the q-th branch in Equation (10) can be obtained from the h−q+2-th eigenvector from the noise subspace.

(13) $$w_{FB,q}=\frac{u_{h-q+2}}{u_{h-q+2,1}}={\left[1,\frac{u_{h-q+2,2}}{u_{h-q+2,1}},\ldots ,\frac{u_{h-q+2,h+1}}{u_{h-q+2,1}}\right]}^{T},q=1,2,\ldots Q,$$

where $u_{h-q+2}={\left[u_{h-q+2,1},u_{h-q+2,2}\ldots ,u_{h-q+2,h+1}\right]}^{T}$ is a $\left(h+1\right)\times 1$ vector. Q<h−r+1 is the number of beamformers. The weight of the q-th multi-tap null-steering beamformer is then obtained

(14) $$w_{q}=-{\left[\frac{u_{h-q+2,2}}{u_{h-q+2,1}},\frac{u_{h-q+2,3}}{u_{h-q+2,1}},\ldots ,\frac{u_{h-q+2,h+1}}{u_{h-q+2,1}}\right]}^{T},$$

The output of the spatial filter is then computed as

(15) $$e_{q}\left[n\right]=z_{F}\left[n-\delta \right]-w_{q}^{T}z_{B}\left[n\right],$$

**Figure 8:**
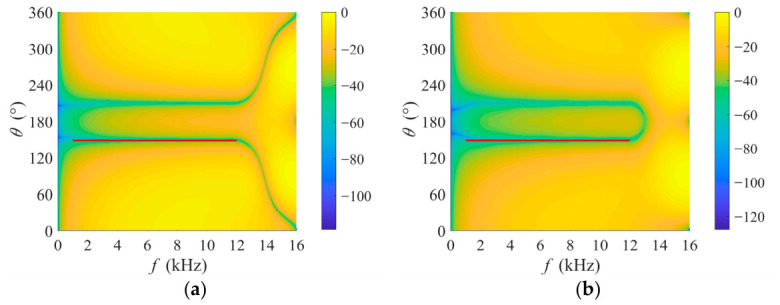
Normalized spatio-temporal amplitude responses of the two branches in the proposed parallel beamformer bank under an interference direction of 150°. The red solid line indicates the interference’s spatio-temporal support. (**a**) The first branch using filter weight $w_{1}$; (**b**) the second branch using filter weights $w_{2}$.

Figure 8 illustrates the normalized spatio-temporal amplitude responses of the two branches in the proposed parallel beamformer bank, where the interference’s spatio-temporal support is indicated by the red solid line. Within the interference’s spatio-temporal support, both branches exhibit a narrower groove comparable to the optimal weight shown in Figure 7b. This reveals that the filter weights constructed from the orthogonal complement of the interference subspace provide reliable interference suppression performance. It is worth noting that each branch demonstrates diverse response patterns outside the interference’s spatio-temporal support region, leading to various cancellations of the target signal across different branches. By fusing the complementary information from the outputs of these branches, effective compensation of the target signal could be achieved. Therefore, this multi-branch parallel design successfully strikes a balance between interference suppression and target signal preservation, thereby overcoming the inherent limitation of traditional unconstrained single beamformer schemes.

### 2.3. The Target Signal Compensation Algorithm for Index Calculation

As is illustrated in Figure 7 and Figure 8, the output spectral gains of the multi-tap null-steering beamformer vary significantly across the bandwidth, violating the flat noise floor assumption required in most acoustic indices. It is essential to integrate additional single-channel noise suppression methods after the denoising preprocessing stage. Since these single-channel noise suppression techniques are relatively mature and fall outside the focus of this paper, they will not be elaborated upon here. This paper takes FADI as a representative index to achieve target signal compensation, since its frequency-dependent threshold is equivalent to single-channel noise suppression to abate the aforementioned influence of uneven spectral gains.

#### 2.3.1. FADI

Given the recording $x\left[n\right]$, the short-time Fourier transform (STFT) is performed to obtain the power spectrogram $P_{x}(k,l),\;1\leq k\leq K,\;1\leq l\leq L$, where k and l index frequency bins and temporal frames. *K* and *L* denote the number of frequency bins and temporal frames, respectively. The binary spectrogram $G_{x}(k,l)$ is then obtained via thresholding,

(16) $$G_{x}(k,l)=\left\{\begin{array}{cc} 1 & P_{x}(k,l)>\eta (k)\\ 0 & \mathrm{otherwise} \end{array}\right.,$$

where the frequency-dependent threshold η(k) for the k-th frequency bin is formulated as

(17) $$\eta (k)=\max\left(\eta_{1},\eta_{2}\left(k\right)\right),\;1\leq k\leq K,$$

where $\eta_{1}=\left(\underset{k_{L}\leq k\leq K,1\leq l\leq L}{\max}P_{x}(k,l)\right)/\varepsilon$ is the single threshold of the conventional ADI. ε is generally 10^5^, and $k_{L}$ is set according to the spectral energy distribution of φ(n) [4]. $\eta_{2}\left(k\right)=\gamma_{1}\cdot Q_{v}\left(k\right)$ is the floating threshold based on constant false alarm rate (CFAR) and $\gamma_{1}$ is the lowest SINR requirement for binarizing each TF point in the power spectrogram. The in-band noise level at the k-th frequency bin can be estimated by

(18) $$Q_{v}\left(k\right)=\frac{1}{L}\sum_{l=1}^{L}P_{v}(k,l),$$

For FADI calculation, the analysis bandwidth is typically divided evenly into I contiguous sub-bands with a bandwidth of 1 kHz each [4]. Let $\vartheta_{i}$ be the index set of frequency bins within the i-th sub-band, i.e., $\vartheta_{i}=\left\{k|(i-1)B\leq (k-1)\Delta f<iB\right\},\;1\leq i\leq I$. FADI is calculated as the Shannon entropy of the normalized spectral energy distribution of biotic sound signals. The energy of virtual species is quantified by the number of non-zero TF points within each sub-band of the binary spectrogram, e.g., $N_{i}=\sum_{k\in \vartheta_{i}}\sum_{l=1}^{L}G_{x}(k,l)$ represents the energy of the i-th virtual species. The relative contribution of the i-th sub-band to the total spectral energy of $G_{x}(k,l)$ is calculated by

(19) $$p_{i}=\frac{N_{i}}{\sum_{j=1}^{I}N_{j}},\;\;1\leq i\leq I,$$

yielding the FADI result

(20) $$FADI=-\sum_{i=1}^{I}p_{i}\ln\left(p_{i}+\xi \right),$$

where ξ is a small, positive number (usually set to 10^−7^) to avoid the logarithm of zero.

#### 2.3.2. The Target Signal Compensation Algorithm

For the output of each filter $e_{q}\left[n\right]$, the corresponding power spectrogram $P_{q}(k,l)$ is first calculated. The binary spectrogram $G_{q}(k,l)$ is then obtained via the frequency-dependent threshold in Equation (17). Afterwards, a point-wise logical OR operation for the above binary spectrograms is conducted, leading to a compensated binary spectrogram, yielding

(21) $$G(k,l)=\vee_{q=1}^{Q}G_{q}(k,l),$$

In such cases, the shared grooves toward directional interference-concentrated bands are retained while mostly compensating the acoustic information of the target signal, thereby mitigating signal self-cancellation effects from these unconstrained adaptive beamformers. Finally, G(k,l) calculated by Equation (21) is forwarded to Equations (19) and (20), leading to the final index result (i.e., BIC-FADI).

## 3. Experiments and Results

This section presents both simulation and real-world experiments to validate the effectiveness of the proposed method. Section 3.1 illustrates the simulation setup. Section 3.2 carries out two simulation experiments across factors, including SINR and target signal preservation. The first experiment is performed under varying SINR options with a fixed acoustic diversity condition. The second one operates in different directions of the target signal when the SINR is −10 dB. Finally, a real-world experiment is conducted in Section 3.3.

To evaluate the performance of the proposed denoising algorithm, we compare the FADI value and its binary spectrogram under different conditions. The standard FADI serves as the baseline without preprocessing. The key performance in preserving the target signal is evaluated by BIC-FADIw, which uses the Wiener–Hopf equation to calculate the filter weights. In contrast, BIC-FADI represents the final output after preprocessing by the complete algorithm illustrated in Figure 2, demonstrating the overall performance gain achieved with the parallel beamformer structure. Here, to investigate the influence of the number of branches on the target signal preservation, the value of *Q* is empirically evaluated ranging from 1 to 5. The resulting indices are referred to as BIC-FADI-*Q* (*Q* = 1, 2, 3, 4, 5), corresponding to different *Q* values.

### 3.1. Simulation Setup

In the simulation experiments, the two microphones form a linear array with an element spacing of 1.06 cm. Without special mention, the speed of sound *c* is 340 m/s, sampling rate *f_s_* = 32 kHz. Since the vocalization frequencies of bird species used in the following experiments are below 12 kHz, we set the maximum frequency for index calculation to 12 kHz to obtain more indicative information in this study. Following the approach in FADI [4], the STFT is implemented using a Hanning window with a frame length of 100 ms and a 100 ms shift. $\gamma_{1}$ = 20 corresponds to a detection probability of 0.9 as well as a false alarm probability of 10^−6^, which is the most commonly used option in practical applications [38]. The parameter settings used in this work are listed in Table 1.

**Figure 9:**
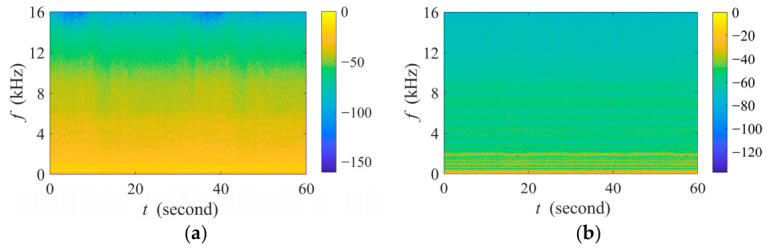
Spectrogram of the 1 min anthropogenic interference signals used in the simulation experiment. (**a**) The aquaculture aerator sound; (**b**) the agricultural drone sound.

In this paper, the experimental recording includes biotic sounds (i.e., avian acoustic event) and acoustic background (i.e., interference source and the noise floor). To emulate avian acoustic events, we generate three types of broadband signals with frequency ranges of 1–12 kHz, 0.5–4 kHz, and 5–9 kHz, respectively, each with a duration of 0.1 s, following the proportional distribution of bird sounds observed in real-world recordings. Anthropogenic interference is represented by recordings of aquaculture aerators and agricultural drones, as shown in Figure 9. Pink noise is used as the noise floor in our experiments, as it represents a fundamental and widespread model of environmental fluctuation across ecological systems [39,40].

It is well known that the SINR is calculated by the ratio between the average power of the target signal and interference-plus-noise. In this work, we multiplied the target signal (i.e., the avian acoustic event) power by a factor *a*_1_ while maintaining the interference-plus-noise power constant, which would result in the desired SINR. Similarly, the desired interference-to-noise ratio (INR) can be obtained by keeping the noise power constant and multiplying the interference power with the corresponding factor *a*_2_. In all simulation experiments, the INR is consistently maintained at 40 dB for each acoustic background. It should be noted that the covariance matrix and cross-correlation vector used in the beamformers are estimated from the interference-plus-noise segments (i.e., the acoustic background) in the experiments described below.

**Figure 10:**
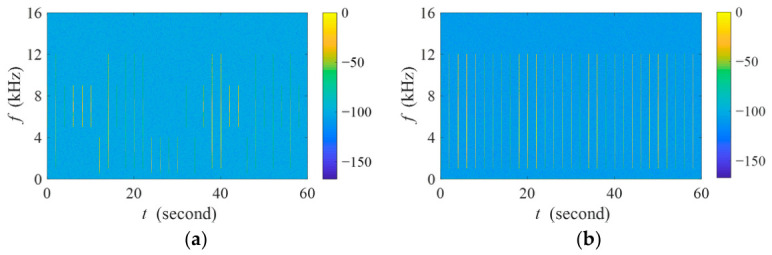
Spectrogram of the 1 min target signals used in the simulation experiments. (**a**) The first experiment; (**b**) the second experiment.

Two simulation experiments are conducted as follows. In the first experiment, each of the two aforementioned interferences is individually overlaid on the pink noise, forming two acoustic backgrounds. For each 1 min acoustic background, 30 acoustic events are individually gain-adjusted and overlaid on it, with each event within every non-overlapping 2 s slot. The first 20 acoustic events are positioned between 0°~90°, and the last 10 are distributed between 150°~180°. The spectrogram of the 1 min target signals used in this experiment is shown in Figure 10a. The interference sources always occur at 150° within the 1 min experimental recording. The experiment is performed over an SINR range from 40 dB to −30 dB in a step size of 5 dB. It should be emphasized that the sequence of events, the place of each event within every non-overlapping 2 s slot, as well as the spatial direction of each event, are fixed to ensure that SINR remains the sole variable. In other words, the acoustic diversity of biotic sound is theoretically the same across all acoustic backgrounds according to the principle of FADI.

The second experiment uses the 1 min acoustic background containing the aquaculture aerator sound as a case study. Here, 30 acoustic events with a duration of 0.1 s and a frequency distribution of 1~12 kHz are used, as shown in Figure 10b. They are overlaid on the aforementioned acoustic background within non-overlapping 2 s intervals at a fixed SINR of −10 dB. To investigate the compensation of the target signal in different directions, the events are divided into six consecutive groups of five. The spatial direction of the first group is set to 180°, and this angle is decreased by 10° for each subsequent group, resulting in a systematic coverage of the azimuth range from 180° to 130°. It is noteworthy that the interference source is placed at 150° throughout the recording.

### 3.2. Simulation Experiment Analysis

#### 3.2.1. The Influence of SINR Variation

**Figure 11:**
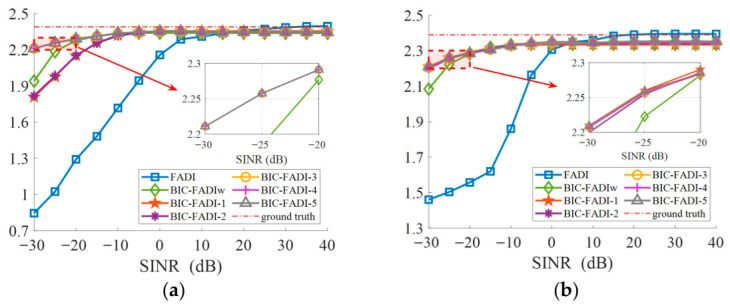
Comparison of FADI, BIC-FADIw, BIC-FADI-1, BIC-FADI-2, BIC-FADI-3, BIC-FADI-4, and BIC-FADI-5 with varying SINRs under different acoustic backgrounds. (**a**) The aquaculture aerator sound; (**b**) the agricultural drone sound. The subplots show a magnified detail of the red boxed area.

Figure 11 presents the comparison of seven indices with varying SINR options under two acoustic backgrounds. In the field of the RBA, for the same acoustic diversity of biotic sound, the effective index should maintain numerical robustness across different acoustic backgrounds and various SINR conditions. It can be observed that compared to the ground truth, extremely small distortion of BIC-FADIw and BIC-FADI family indices (i.e., BIC-FADI-1, BIC-FADI-2, BIC-FADI-3, BIC-FADI-4, and BIC-FADI-5) exists under high SINR conditions. This is because the array signal processing suppresses ten acoustic events near the interference direction. It is worth noting that the minor distortion falls within the acceptable range for ecoacoustic interpretation and does not compromise the validity of conclusions established in the existing literature.

It is obvious that multi-channel denoising preprocessing methods (e.g., BIC-FADIw and BIC-FADI family indices) are superior to the single-channel one (e.g., FADI) when several acoustic backgrounds are considered. Specifically, the multi-channel denoising preprocessing methods provide a significantly robust performance within a wide SINR range from −10 dB to 40 dB. The fundamental reason for this advantage lies in the higher system degrees of freedom provided by the employed multi-tap beamformer, which enables superior broadband interference suppression. As a result, the SINR level can meet the requirements for subsequent FADI calculations.

It is important to highlight that under extremely low SINR conditions (e.g., −30 dB~−20 dB), the BIC-FADI family indices not only outperform the single-channel FADI but also exhibit a slower value degradation than BIC-FADIw. This robustness advantage is rooted in the design of the multi-branch parallel beamformer, which leverages complementary information fusion to preserve avian vocalization details that are typically compromised in unconstrained single filter designs.

For any SINR condition, the values of BIC-FADI-3, BIC-FADI-4, and BIC-FADI-5 in Figure 11a differ by less than 0.002, while those of BIC-FADI-1~BIC-FADI-5 in Figure 11b differ by less than 0.1. In summary, BIC-FADI-3, BIC-FADI-4, and BIC-FADI-5 provide similar performance, which implies that *Q* = 3 is a suitable choice.

**Figure 12:**
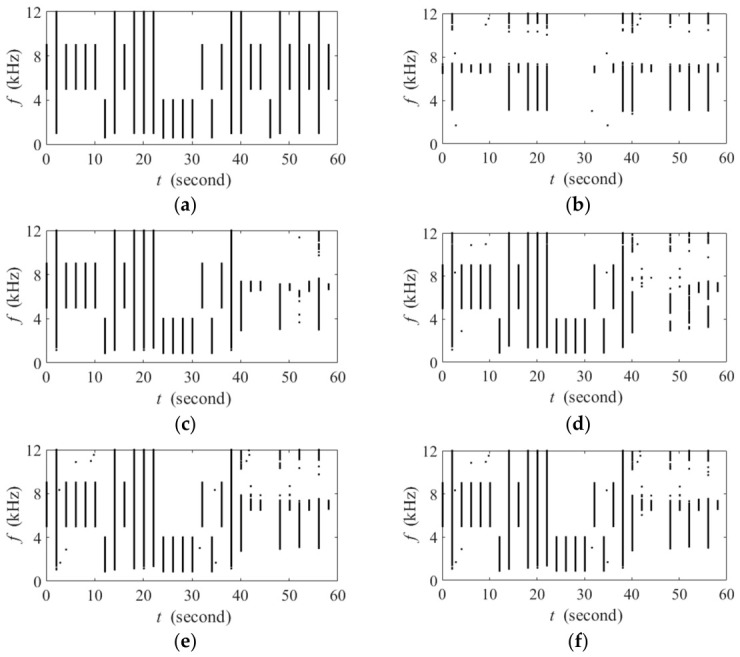
A case study under an aquaculture aerator background at −10 dB SINR. (**a**) The binary spectrogram of ground truth; (**b**) the binary spectrogram of FADI; (**c**) the binary spectrogram of BIC-FADIw; (**d**) the binary spectrogram of BIC-FADI-1; (**e**) the binary spectrogram of BIC-FADI-3; (**f**) the binary spectrogram of BIC-FADI-5.

Figure 12 presents a case study under the aquaculture aerator background at −10 dB SINR, providing a definitive verification of our proposed method’s interference suppression capability and the biotic sound preservation performance. Compared with the ground truth in Figure 12a, many missed detections (corresponding to weak biotic sound-dominated TF points below the threshold) had spread in the binary spectrogram of FADI (Figure 12b). In contrast, the binary spectrograms of BIC-FADIw (Figure 12c) and BIC-FADI family (Figure 12d–f) indices show significantly better preservation of biotic sound than FADI, which implies that the null-steering beamformer successfully improves the SINR of the noisy recordings.

Thanks to the parallel beamformer structure used in BIC-FADI, it achieves an optimal balance between interference suppression and biotic sound information preservation. The performance variation between BIC-FADIw and BIC-FADI family indices is largely due to differences in preserving biotic sounds near the interference direction. In the simulation, the last ten acoustic events between 40 s and 60 s are in the vicinity of the angle of the interference source. A comparison of the distribution of these ten acoustic events in the binary spectrograms reveals distinct behaviors. BIC-FADIw detects biotic sound-dominated TF points mainly concentrated in the 4–8 kHz range (Figure 12c). In contrast, BIC-FADI-*Q* (Figure 12d–f) not only detects more TF points in the high-frequency range (e.g., 10–12 kHz) but also retains more biotic sound information in the mid-frequency range, such as around 52 s. This advantage enhances the ability of acoustic indices to reflect biotic information in complex environments.

#### 3.2.2. The Performance of Target Signal Preservation

**Figure 13:**
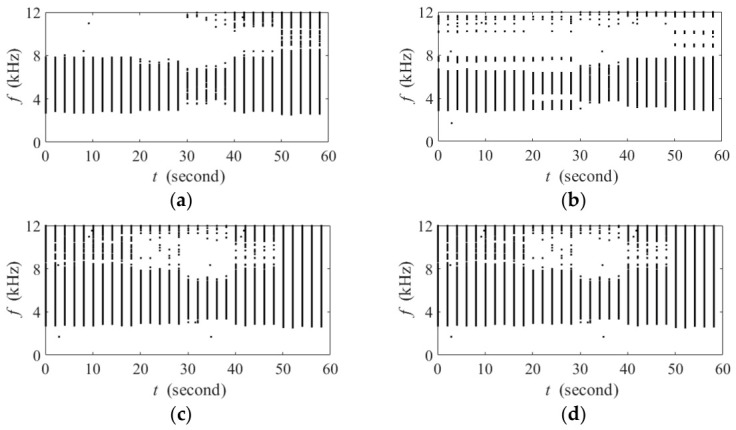
Comparison of target signal preservation under aquaculture aerator background at −10 dB SINR. (**a**) The binary spectrogram of BIC-FADIw; (**b**) the binary spectrogram of BIC-FADI-1; (**c**) the binary spectrogram of BIC-FADI-3; (**d**) the binary spectrogram of BIC-FADI-5.

Figure 13 presents the performance of the target signal preservation from BIC-FADIw and three BIC-FADI family indices (i.e., BIC-FADI-1, BIC-FADI-3, and BIC-FADI-5) under the aquaculture aerator background when SINR = −10 dB. In this experiment, acoustic events from 0 to 10 s are located at 180°, those from 10 to 20 s at 170°, and so on. Accordingly, the acoustic events from 30 to 40 s correspond to biotic sounds arriving from the same direction as the interference source (i.e., 150°). For BIC-FADIw, when the biotic sounds arrive from directions ranging between 180° and 150°, many TF points corresponding to biotic sounds in high-frequency are missed, with the most severe missed detections observed at 150°. As for BIC-FADI-*Q*, although *Q* = 1 also leads to many missed detections in the high-frequency range (Figure 13b), increasing *Q* enables complementary contributions from different parallel branches. Consequently, the TF points corresponding to biotic sounds from directions away from the interference source (e.g., 180°, 170°, and 130°) are effectively preserved (Figure 13 c,d). Moreover, the number of the biotic sound-dominated TF points near the interference source (140° and 160°) increases significantly. Even for the interference direction (150°), the TF points associated with biotic sounds in the low-frequency range (below 4 kHz) and high-frequency range (above 10 kHz) are also preserved to some extent. The results indicate that biotic sounds originating from 130° to 180° (except the 150° interference direction) are suppressed in BIC-FADIw but are preserved in the BIC-FADI-*Q* indices. This implies that our proposed parallel beamformer structure effectively reduces the self-cancellation of the target signal, especially for biotic sounds near the interference direction.

While the amount of preserved acoustic information generally increases with the value of *Q*, the performance plateaus beyond *Q* = 3 in this experiment, which is consistent with the results shown in Figure 11. At this value, the acoustic information is already retained with relative completeness. Consequently, any further increase in *Q* fails to yield a significant performance gain, only introducing a higher computational burden. This conclusion provides critical insight into the numerical relationship between the interference subspace dimension r and the filter length h. The saturation of performance at *Q* = 3 suggests an optimal operational point. Specifically, it implies that setting the filter length to be at least five taps greater than the estimated interference subspace dimension (i.e., h>r+5) is a prudent design rule. This rule ensures that the smallest three eigenvectors can be reliably attributed to the noise subspace, thereby reducing the probability of false alarms arising from residual interference components.

### 3.3. The Real-World Experiment

**Figure 14:**
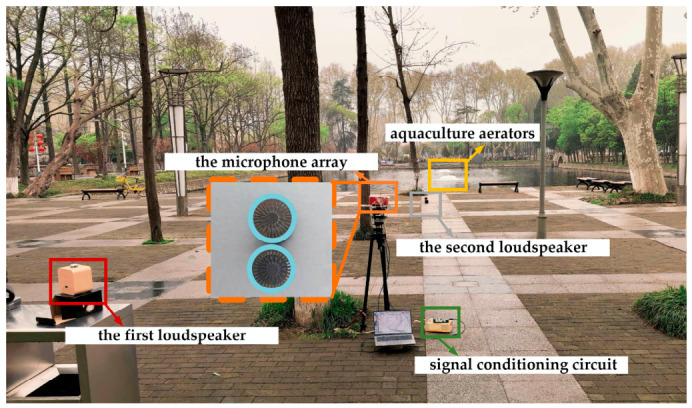
The real-world experimental deployment. The microphone array, the signal conditioning circuit, the first loudspeaker, the second loudspeaker, and the aquaculture aerator interferences are marked by orange, green, red, gray, and yellow boxes, respectively. The recording is collected by the two microphone sensors spaced 1.4 cm apart, with their specific locations marked by blue circles.

**Figure 15:**
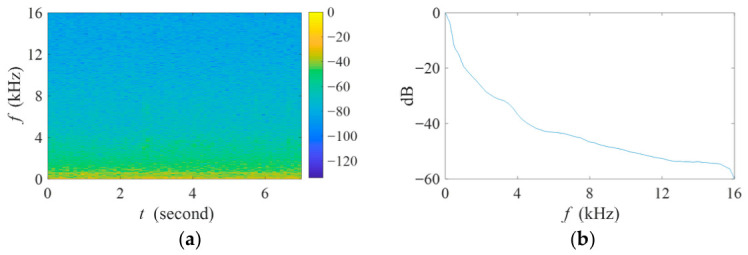
(**a**) Spectrogram and (**b**) normalized power spectrum of the interference-plus-noise signal used in the real-world experiment.

In the real-world experiment, a custom-made two-element acoustic recording device, consisting of two microphone sensors spaced 1.4 cm apart, was placed near the pond as shown in Figure 14. Specifically, the microphones (Model: 378C20) and signal conditioning circuit (Model: UA326Hi) were deployed accordingly. The geometry of the real-world experiment is illustrated in Supplementary Figure S1. The distances of all sources were measured by a laser rangefinder, and their azimuth angles were obtained through prior measurements using a theodolite. Here, an acoustic background with two aquaculture aerators was selected, with respective angles of 178° and 183° relative to the microphone array axis, and distances of 79 m and 109 m from the array. The aerators were continuously operated, and the spectrogram and normalized power spectrum of this interference-plus-noise signal are shown in Figure 15. It is worth noting that the filter weights are calculated based on these interference-plus-noise segments.

**Figure 16:**
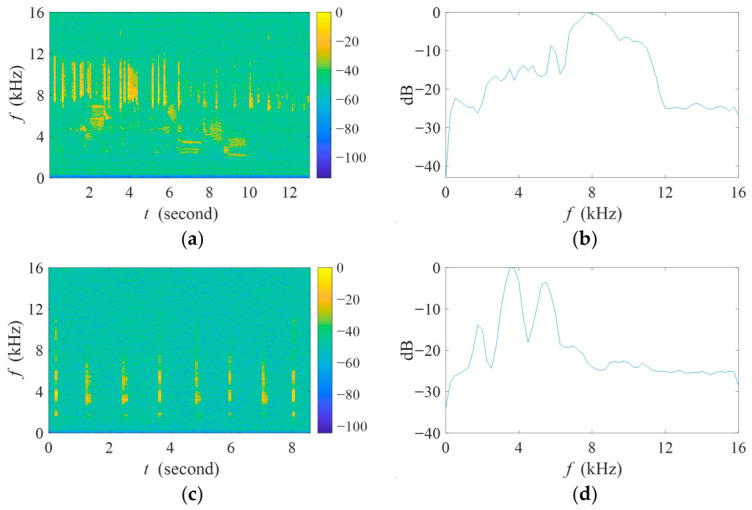
(**a**) Spectrogram and (**b**) normalized power spectra of two real avian recordings used in the real-world experiment. (**a**) The spectrogram of the length-13s real avian recording; (**b**) the normalized power spectrum of the length-13s real avian recording; (**c**) the spectrogram of the length-9s real avian recording; (**d**) the normalized power spectrum of the length-9s real avian recording.

To quantitatively evaluate the interference suppression capability and the biotic sound preservation performance of the proposed algorithm, two real avian recordings were cyclically played from two loudspeakers. The first loudspeaker cyclically played the length-13s real avian recording during the initial 60 s (0–60 s), which was positioned at an angle of 20° and a distance of 1.8 m from the array. The second loudspeaker, placed at an angle of 171° and a distance of 8.2 m, played the length-9s real avian recording during the subsequent 40 s (60 –100 s). Note that the second loudspeaker was in the vicinity of the interference sources’ direction. The spectrograms and normalized power spectra of the two original avian recordings are provided in Figure 16.

**Figure 17:**
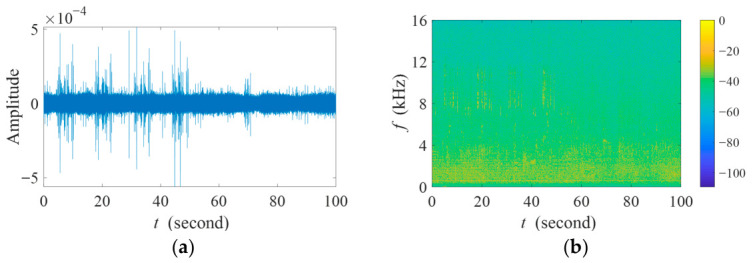
(**a**) Waveform and (**b**) spectrogram of the length-100s field recording.

Figure 17 shows the length-100s field recording collected by the reference channel. It is worth noting that the recorded length-100s mixture also contained spontaneous local bird vocalizations. Therefore, the received target signal is not identical to the original playback recordings. The overall SINR of the recording is merely −1.73 dB.

**Figure 18:**
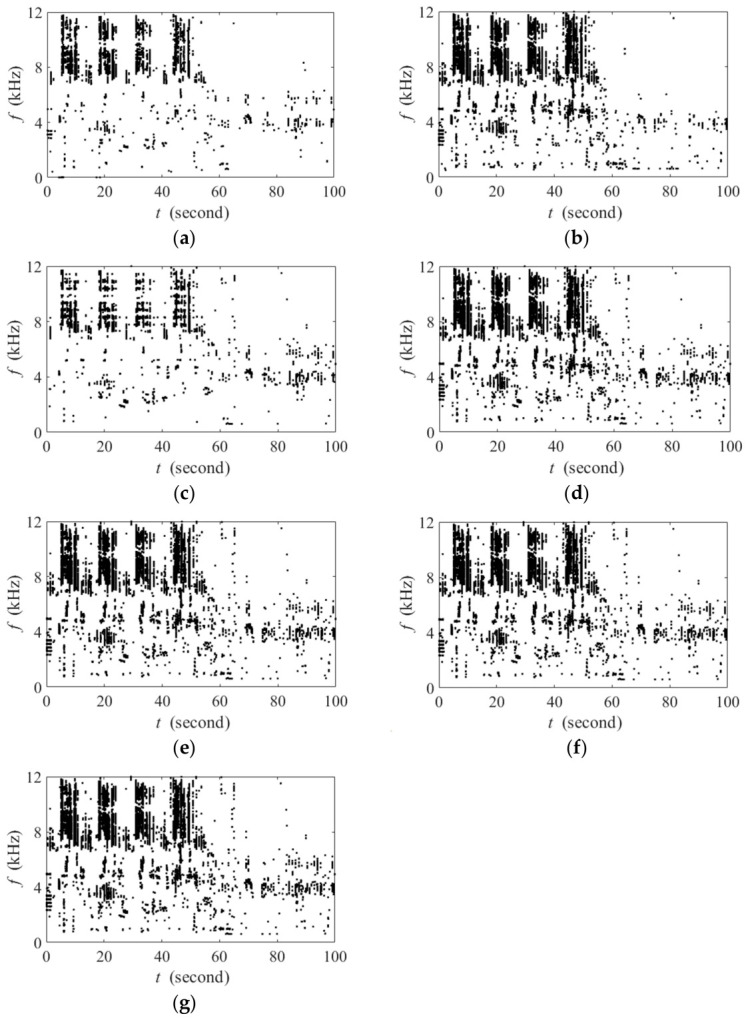
The binary spectrograms of the length-100s field recording. (**a**) FADI; (**b**) BIC-FADIw; (**c**) BIC-FADI-1; (**d**) BIC-FADI-2; (**e**) BIC-FADI-3; (**f**) BIC-FADI-4; (**g**) BIC-FADI-5.

In the real-world experiment, the filter length is set to 51, and the delay item is 25 according to the subspace dimension of interference, while other parameter settings are the same as the simulations. As shown in Figure 18a, the frequency-dependent thresholds of FADI effectively suppress the influence of interference and ambient noise. However, strong interference noise also causes some TF points associated with weak avian vocalizations to fall below the corresponding thresholds, resulting in considerable missed detections. Specifically, the biotic sound components within the 1–7 kHz range between 0 s and 85 s are almost completely masked in the binary spectrogram.

In comparison with FADI, Figure 18b–g show that multi-channel denoising preprocessing methods significantly improve the SINR of the recording, thereby considerably reducing missed detections in the binary spectrograms. This is in line with the simulation experiments. However, as demonstrated in Section 3.2, BIC-FADIw suffers from the self-cancellation of biotic sounds, especially for those near the interference direction. As is illustrated in Figure 18b, biotic sound at 2–4 kHz between 35 s and 40 s has been suppressed, resulting in missed detections. Even worse, when the second loudspeaker played the second real avian recording in the vicinity of the aerators’ direction, certain TF points associated with avian vocalizations failed to exceed the frequency-dependent threshold, causing numerous missed detections in the later segment of the recording (e.g., biotic sound at 2–6 kHz between 60 s and 100 s).

It is important to emphasize that, as is shown in Figure 18c–g, the proposed method achieves deep interference suppression while preserving the most biotic sound TF information as expected. It should also be mentioned that *Q* = 3 suggests an optimal operational point, which is in line with our simulation experiment. For the field scenario with two aerators, the proposed multi-tap null-steering beamformer can dynamically track real-time variations in interference noise within field recordings, effectively preventing most interference-dominated TF points from appearing in the binary spectrogram. Simultaneously, the proposed parallel beamformer structure compensates for the TF information loss of the biotic sounds caused by the unconstrained single filter.

## 4. Discussion

In practical applications, the SINRs of biotic sounds in highly complex acoustic environments are often lower than practitioners expect. This makes it difficult for existing commonly used acoustic indices to correctly reflect the acoustic complexity and diversity of biotic sounds under low SINR conditions [4]. In this work, the denoising preprocessing approach prior to acoustic indices calculations was proposed to improve their robustness.

As shown in Section 3, both simulation and real-world experiments confirmed that the proposed approach achieves deep interference suppression while maximizing the preservation of avian vocalizations within a much wider SINR range in urban–rural soundscapes characterized by directional anthropogenic interference. These capabilities are particularly desirable for conservation biologists who employ acoustic sensors for long-term ecosystem monitoring, as they enable the reliable remote sensing of ecological acoustic information in challenging urban–rural areas. Beyond acoustic index calculations focused on this study, the proposed denoising preprocessing framework can also serve as a general-purpose front-end module for acoustic event detection and species recognition tasks within environmental sensor networks.

This work focuses on the denoising preprocessing before acoustic indices calculations in the urban–rural area, where a single dominant interference source originates from a narrow angular sector. However, as RBA increasingly extends into urban soundscapes due to ongoing urbanization, the acoustic environment becomes significantly more complex. Here, there are many types of sounds associated with human activities, such as human speech, traffic noise (from cars, airplanes, etc.), and mechanical noise (from lawn mowers, construction machinery, etc.) [41]. Critically, these multiple interference sources often arrive from arbitrary and dynamic spatial directions. Therefore, an important future direction lies in extending the current framework to address the challenges posed by fast-moving and multiple dispersed interferences, aiming to improve the robustness of acoustic indices in highly complex acoustic environments via advanced signal processing techniques.

It is also worth noting that a dual-microphone array was chosen in this study because large-scale, long-term biodiversity monitoring requires simple and low-cost hardware. However, the complete preprocessing framework is inherently generalizable. It involves first constructing beams towards both the desired signal and the interference directions, followed by an interference suppression stage and a subsequent signal compensation algorithm. This principle can also be realized using other existing acoustic recording devices, such as NT-SF1 Ambisonic systems and Song Meter recorders. This generality underscores the broader applicability of the proposed preprocessing framework, extending well beyond the specific hardware employed in this work.

## 5. Conclusions

This paper proposes a general-purpose denoising preprocessing algorithm before acoustic indices calculation to enhance their robustness in complex urban–rural soundscapes. The integration of our preprocessing method with FADI not only serves as a validation but also gives rise to a novel and more superior index for application in noisy environments, i.e., BIC-FADI. The workflow of the proposed method is provided. First, an acoustic recording device consisting of two close-spaced microphone sensors is employed to construct a FO-DMA, followed by a parallel bank of multi-tap null-steering beamformers. By constructing the filter weights from the noise subspace, each beamformer in the bank forms a deep groove within the interference’s spatio-temporal support while exhibiting diverse and complementary response patterns to the target signals. Finally, leveraging the inherent differences in target signal preservation among the branches, a point-wise logical OR fusion algorithm is applied to the binary spectrograms from each output. Simulation results confirm that BIC-FADI can maintain numerical robustness over a much wider SINR range from −10 dB to 40 dB, which is superior to FADI. The real-world experiment also shows that the binary spectrogram of the proposed method achieves deep interference suppression while maximizing the preservation of avian vocalizations. We propose that BIC-FADI is a good candidate for reliable RBA applications in urban–rural soundscapes.

## Figures and Tables

**Table 1:** Parameter settings used in this work.

Parameters	Values
delay item: δ	10
filter length h	21
sampling frequency: *f_s_*	32 kHz
number of frequency bins: *K*	1200
number of temporal frames: *L*	600
number of sub-bands: *I*	12
parameter of single threshold in ADI:$k_{L}$	20
parameter of floating threshold:$\gamma_{1}$	20
number of branches: *Q*	(1, 2, 3, 4, 5)

## Data Availability

The data can be obtained from the corresponding author upon reasonable request.

## Supplementary Materials

The following supporting information can be downloaded at: https://www.mdpi.com/article/10.3390/s26103186/s1, Figure S1: The geometry of the real-world experiment.
