# Supplementary figures and images for: A Denoising Preprocessing Framework via Orthogonal Multi-Tap Null-Steering Beamformer Bank: Facilitating Target Signal Preservation Under Low SINR Conditions and Complex Soundscapes
Source: Sensors (Basel). 2026 May 18;26(10):3186. doi: 10.3390/s26103186 (PMC13210416; doi:10.3390/s26103186)

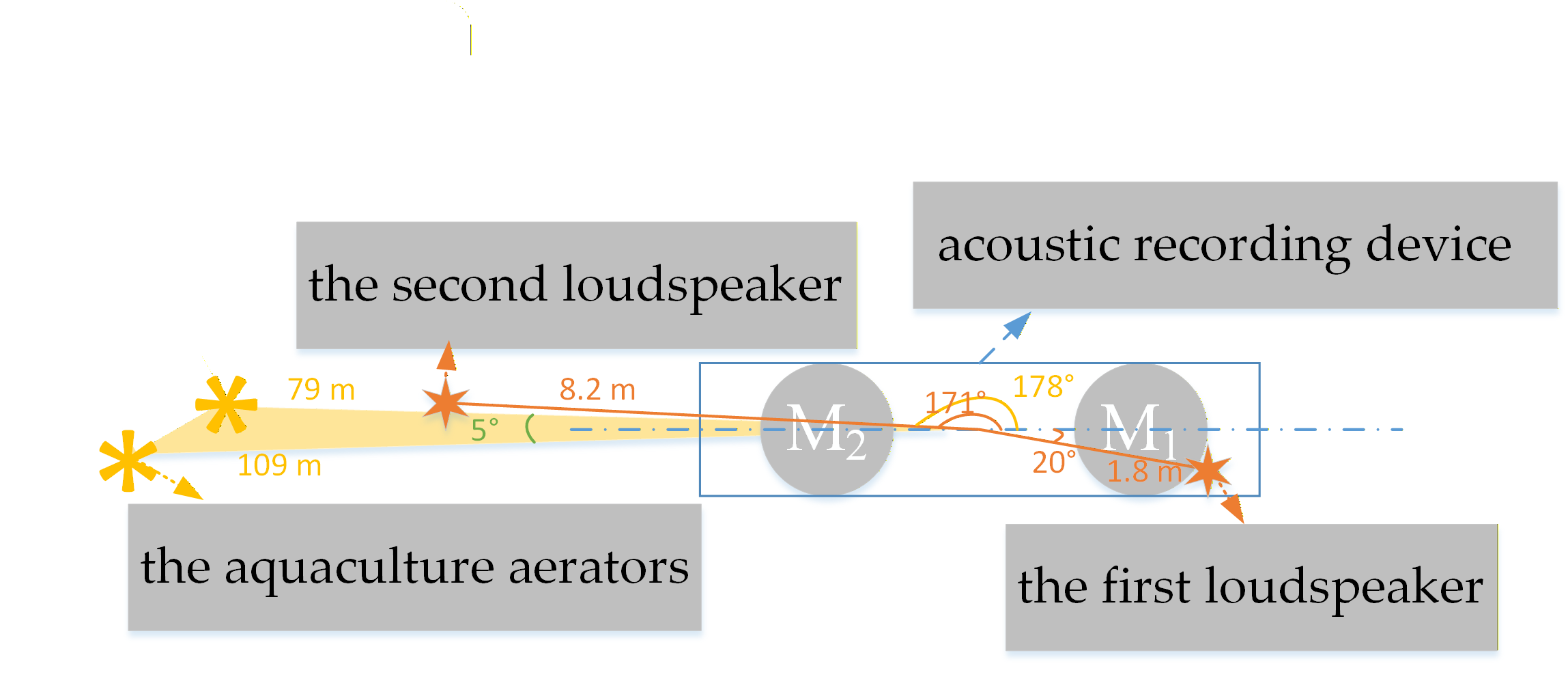

Supplement: Supplementary file 1 [file sensors-26-03186-s001.zip › sensors-4112956-supplementary Figure S1.png]
